# Diverse Reservoirs of Third-Generation Cephalosporin-Resistant *Escherichia coli* in Guatemala

**DOI:** 10.1007/s10393-026-01775-9

**Published:** 2026-01-23

**Authors:** Gabriela Perez-Garcia, Danilo Alvarez, Andrea Gomez Barillas, Renata Mendizabal-Cabrera, Brooke M. Ramay, Tin Ho, Nikolina Walas, Jay P. Graham

**Affiliations:** 1 Department of Epidemiology and Biostatistics, School of Public Health, University of California, 2121 Berkeley Way, Berkeley, CA 94704 USA; 2 Centro de Estudios en Salud, Universidad del Valle de Guatemala, Guatemala City, Guatemala; 3 Paul G. Allen School for Global Health, Washington State University, Pullman, WA USA; 4 Environmental Health Sciences Division, School of Public Health, University of California, Berkeley, CA USA

**Keywords:** one Health, antibiotic resistance, Guatemala, *Escherichia coli*, third-generation cephalosporin resistance, extended-spectrum β-lactamases

## Abstract

In Guatemala, there is a significant gap in our understanding of the prevalence of antibiotic resistance across different reservoirs likely to affect human exposures. We conducted a One Health study of produce, poultry, water, and humans within peri-urban and urban Guatemalan markets to characterize the prevalence of third-generation cephalosporin-resistant *Escherichia coli* (3GCR-EC). We screened a total of 132 samples for 3GCR-EC using selective media. Samples were collected across three markets in Guatemala and included produce (*n* = 32), public tap water (*n* = 30), commercial and non-commercial poultry (*n* = 40), and human fecal matter (*n* = 30). We measured phenotypic and genotypic resistance using culture-based methods and whole-genome sequencing. Among the 132 samples, 87.1% (*n* = 115) were positive for *E. coli*, and 67.4% (*n* = 43) were positive for 3GCR-EC. After removing duplicates, a total of 40 3GCR-EC isolates were sequenced from commercial chicken carcasses (*n* = 8), non-commercial chicken carcasses (*n* = 16), water (*n* = 4), and human feces (*n* = 12). Out of 11 unique extended-spectrum beta-lactamase genes, the most prevalent gene was *bla*_CTX-M-55_. There was high clonal diversity, with 32 distinct sequence types (ST) detected. Clinically important pandemic lineages of *E. coli* were identified: ST10, ST648, and ST410. This study identified a high prevalence of antibiotic resistance across diverse samples within public Guatemalan markets. The frequent detection of 3GCR-EC, their resistance genes, and epidemiologically important lineages of *E. coli* identified from humans, produce, water, and poultry samples highlight the importance of implementing more extensive surveillance using a One Health approach to develop coordinated strategies to mitigate the spread of antibiotic resistance.

**Supplementary Information:**

The online version contains supplementary material available at 10.1007/s10393-026-01775-9.

## Introduction and Purpose

Antibiotic resistance (ABR) is a growing threat to public health. Although projections differ by source, some have estimated that 10 million people will die of antibiotic-resistant infections every year by 2050 (O’Neill, 2014). The drivers of ABR are varied, but non-clinical drivers, including the misuse of antibiotics in livestock production and human medicine, as well as the lack of human and animal wastewater collection and treatment, are important factors in the rising prevalence of ABR (Martin et al., 2015).

*Escherichia coli* (*E. coli*) is a fecal indicator bacterial species that is widespread in humans, animals, and the environment (Leonard et al., 2018). Though *E. coli* can be a commensal organism, there are certain pathotypes, including diarrheagenic *E. coli* (DEC) and extraintestinal pathogenic *E. coli* (ExPEC), that primarily cause most extraintestinal diseases, including urinary tract and bloodstream infections in humans (Russo & Johnson, 2000). There are many ExPEC strains, but certain lineages characterized by ExPEC sequence types, such as ST131 and ST10, are commonly found when genotyping *E. coli* from extraintestinal infections (Manges et al., 2019). These ExPEC sequence types are known as pandemic lineages because of their global abundance and frequency in causing disease.

Resistance in *E. coli* populations to extended-spectrum beta-lactam drugs has been increasing and is largely mediated by resistance genes known as extended-spectrum beta-lactamases (ESBLs). The spread of these resistance genes can be complex, through both vertical and horizontal gene transfer, and ESBL-producing *E. coli* (ESBL-EC) are typically resistant to broad-spectrum antibiotics, including penicillin and cephalosporins (Hussain et al., 2021; Knothe et al., 1983). ESBL-EC is commonly used as an indicator in ABR national surveillance programs (Anjum et al., 2021). ESBL-EC has been considered a serious threat by the US Centers for Disease Control and Prevention (CDC), which has introduced programs, such as the Multi-site Gram-negative Surveillance Initiative, to surveil ESBL-Enterobacterales (Duffy et al., 2023). The World Health Organization has also initiated the Tricycle protocol in 2018 to surveil ESBL-EC infections in humans, livestock, and water (WHO Integrated Global Surveillance on ESBL-Producing E. coli Using a One Health Approach, 2021). Third-generation cephalosporin-resistant *E. coli* (3GCR-EC) is an emerging dominant phenotype and has been associated with more severe clinical outcomes and has been shown to increase the risk of mortality (Camilleri et al., 2023; Pitout & Laupland, 2008).

A “One Health” approach focuses on the study of disease arising from the interaction between humans, the environment, and animals (Mackenzie & Jeggo, 2019). A benefit of this framework in ABR surveillance is the potential to elucidate exposure pathways and assess the risk of particular reservoirs of ABR (Lebov et al., 2017). Previous literature has shown that limited studies are using the One Health framework. In a systematic review of drug-resistant *E. coli* in India, only two of the 38 studies assessed all three aspects of the One Health framework (Rajagopal et al., 2021). In Central America, most studies have focused primarily on human isolates (O’Neal et al., 2020). Evidence on the importance of the environment in the spread of AMR is growing, but gaps remain (Fletcher, 2015).

In Guatemala and other Central American countries, there are currently no studies to our knowledge that have investigated multiple reservoirs of ABR, including humans. Additionally, it is unknown which sectors, such as livestock and poultry production, may play a key role in the spread of ESBL-EC in Guatemala, which is the largest broiler chicken-producing country in Central America (Jarquin et al., 2015). In this study, we investigated the prevalence of 3GCR-EC and assessed genotypic and phenotypic resistance. We hypothesized that human and non-human 3GCR-EC would be highly related in terms of the ABR genes and sequence types circulating.

## Methods

### Study Area and Design

We conducted a cross-sectional study utilizing randomly selected convenience samples at three Guatemalan markets located less than 44 miles from the Universidad del Valle de Guatemala (UVG). The markets were in Ciudad de Guatemala, Guatemala; Santa Catarina Pinula, Guatemala; and San Martín Jilotepeque, Chimaltenango.

This study involved environmental and human samples collected from two peri-urban Guatemalan markets and one urban market, from June to August 2022. In order to assess the prevalence of ABR across different reservoirs of interest, the following sources were sampled: non-commercial poultry, commercial poultry, produce, soiled toilet paper from bathroom stalls, and tap water. The final sample consisted of non-commercial chicken carcasses (*n* = 20), commercial chicken carcasses (*n* = 20) sold by vendors, produce (strawberries, cilantro, watercress, lettuce) (*n* = 32) sold by vegetable and fruit vendors, toilet paper used for anal cleansing, found in the trash receptacles of market bathrooms (*n* = 30), and public tap water samples from the markets (*n* = 30). Non-commercial chicken carcasses were categorized as those sold without packaging and without an identifiable brand. We used toilet paper samples as a proxy for human fecal samples and to estimate the prevalence of antimicrobial resistance within the human population.

### Sample Collection

A randomly generated list of market stalls that sold produce or poultry was created using the RAND function in Excel. This list assigned a market stall for all produce samples and a market stall for all poultry samples that would be visited on each sampling day. If the assigned market stall or the products that needed to be purchased were not available, the next market stall on the list was used. Water samples were collected from publicly available tap water sources using Aquagenx® sampling bags according to the manufacturer’s instructions (Young et al., 2022). Pieces of toilet paper where fecal matter was observed were collected from the bathroom stall trash bins within the markets. Stalls in each bathroom were sampled for toilet paper once per collection day to ensure that no samples were duplicated (Perez-Garcia et al., 2024). The soiled toilet paper was aseptically placed in a sterile Whirl–Pak® bag (55 oz) after collection. All samples were transported to UVG in coolers using ice packs (4–8°C) and analyzed within 6 h of collection.

### Detection of *Escherichia coli*

For commercial and non-commercial poultry samples, whole chicken carcasses were transferred from the sample collection bag into a Whirl–Pak® bag (55 oz). Four hundred milliliters (mL) of peptone buffer was added to the bag and was then shaken at 200 rotations per minute (rpm) for 15 min at room temperature (20–22°C) (Jarquin et al., 2015). After the shaking was complete, the peptone buffer was poured from the bag into three 50-mL conical tubes, each tube having approximately the same volume of the solution. Ten grams of the produce sample was measured on a laboratory balance and then placed into a Whirl–Pak® bag (55 oz). Peptone buffer (90 mL) was added to the bag and shaken for 15 min at 37°C (Montero et al., 2021). Next, the peptone buffer was poured from the bag and distributed evenly into two 50-mL conical tubes. For water sample processing, two milliliters from each positive compartment of the Aquagenx® bag were collected using a disposable pipette. The media were transferred to a 50-mL conical tube.

The tubes with the solution collected from the poultry, produce, and water samples were centrifuged at 5000 rpm for 10 min at 20°C. The supernatant was disposed from each tube, and the bacterial pellet was resuspended using 500 µL (µL) of Tryptic Soy Broth medium (Difco, USA). The media from the tubes respective to each sample were combined into one 50-mL conical tube and then vortexed for 5 s to prepare for culturing. The media was then streaked onto MacConkey agar (Difco, Maryland) absent of antibiotics and incubated at 37°C for 24 h, after which ten lactose-positive colonies appearing dark pink or red were selected from each MacConkey plate for ESBL testing (Lautenbach et al., 2008).

Toilet paper samples were removed from the Whirl–Pak® sampling bag (55 oz) in a biosafety cabinet. A sterile cotton swab saturated with Tryptic Soy Broth medium (Difco, USA) was rubbed on the areas of the toilet paper with the most observed fecal matter and streaked onto a MacConkey plate (Difco, Maryland) and incubated at 37°C for 24 h using the same microbiology protocols as above (Perez-Garcia et al., 2024).

### Isolation of 3GCR-EC

Ten putative *E. coli* colonies selected from the MacConkey plate were plated on CHROMagar™ ESBL and incubated at 37°C for 24 h (Hornsey et al., 2013). Up to two dark pink or red isolates were identified as putative 3GCR-EC and selected from plates. Isolates were frozen at −80°C in Tryptic Soy Broth medium (Difco, USA) with 15% glycerol. We used a negative control (non-ESBL-producing *E. coli*, ATCC 25922) and a positive control (ESBL-producing *E. coli*, ATCC 13476) when culturing each batch of samples for quality assurance.

### DNA Sequencing and Bioinformatic Analysis

Genomic DNA sequence analysis was completed using the following methods. Briefly, DNA from the selected isolates was extracted using the DNeasy Blood and Tissue kit (Qiagen, Hilden, Germany) according to the manufacturer’s instructions. Whole genome sequencing data from the isolates were generated using an Illumina NovaSeq 6000 platform with a paired-end protocol (Nextera XT library; Illumina) to create ~150-bp length reads. The quality of the reads was assessed using FastQC version 0.11.9 (Babraham Institute, 2019). De novo assemblies of the paired short reads were generated using Unicycler version 0.4.9 (Wick et al., 2017). Computation was carried out using the Savio high-performance cluster (HPC) provided by the University of California, Berkeley. Assembled genome sequences were evaluated based on quality metrics using QUAST version 5.2 (Mikheenko et al., 2018). Contigs below 500 bp were excluded from the final draft assemblies.

Antibiotic resistance genes were identified using the ABRicate tool version 1.0 (Zankari et al., 2012). The ResFinder database, one of the pre-downloaded databases in ABRicate, was used to detect resistance genes in the sequences. The assembled sequences were then genotyped using multilocus sequence typing (MLST) with the MLST package version 2.23.0 (https://github.com/tseemann/mlst) to identify unique clonal groups based on highly conserved bacterial genes (Jolley et al., 2018). Isolates determined to have identical MLST and ARG profiles and that originated from the same sample as another isolate were considered duplicates and excluded from the analysis.

Data visualization and cleaning was conducted using the R programming language (R Core Team, 2021). The R package ‘ggplot2’ was used to visualize the presence of beta-lactamase genes among the isolates.

PHYLOViZ 2 was used to generate the phylogeny tree, utilizing the goeBURST Full MST algorithm (Feil et al., 2004; Francisco et al., 2009, 2012; Nascimento et al., 2017). To improve readability and appearance, a slight visual touch-up of the diagram was performed using the GNU Image Manipulation Program (GIMP).

## Results

A total of 132 samples were collected and analyzed during the study period from June to August 2022. One hundred and fifteen samples (87.1%) were positive for putative *E. coli*, including 50% of water samples and 93.8% of produce samples. Forty-three samples (32.6%) tested positive for 3GCR-EC. A total of eighteen non-commercial poultry samples (90%), nine commercial poultry samples (45%), one produce sample (3.1%), ten human samples (33.3%), and five water samples (16.7%) tested positive for 3GCR-EC (Table 1).

**Table 1:** Distribution of *E. coli* and third-generation cephalosporin-resistant *E. coli* (3GCR-Ec) positivity by sample type and market, among environmental samples from peri-urban and urban markets in Guatemala City, Guatemala.

	*E. coli*-negative *n* (%)	*E. coli*-positive *n* (%)	3GCR-Ec-negative *n* (%)	3GCR-Ec-positive *n* (%)
Total (*n* = 132)	17 (12.9)	115 (87.1)	89 (67.4)	43 (32.6)
*Sample type*				
Non-commercial poultry (*n* = 20)	0 (0.0)	20 (100.0)	2 (10.0)	18 (90.0)
Commercial poultry (*n* = 20)	0 (0.0)	20 (100.0)	11 (55.0)	9 (45.0)
Produce (*n* = 32)	2 (6.2)	30 (93.8)	31 (96.9)	1 (3.1)
Human (*n* = 30)	0 (0.0)	30 (100.0)	20 (66.7)	10 (33.3)
Water (*n* = 30)	15 (50.0)	15 (50.0)	25 (83.3)	5 (16.7)
*Market*				
San Martín Jilotepeque (*n* = 46)	0 (0.0)	46 (100.0)	29 (63.0)	17 (37.0)
Guatemala City (*n* = 10)	0 (0.0)	10 (100.0)	5 (50.0)	5 (50.0)
Santa Catarina Pinula (*n* = 76)	17 (22.4)	59 (77.6)	55 (72.4)	21 (27.6)

Seventy-two isolates were identified from 43 samples positive for 3GCR-EC. Sixty-nine isolates (96%) were successfully sequenced, and 40 isolates remained after the removal of duplicates. The sample type distribution of the isolates positive for at least one ESBL gene consisted of 8 out of 20 commercial chicken carcass samples, 16 out of 20 non-commercial chicken carcass samples, 4 out of 30 water samples, 12 out of 30 human samples, and no produce samples (see Supplemental Table S1).

### Prevalence and Distribution of ESBL Genes Among Reservoirs

There was variation in the number and type of beta-lactamase (*bla*) genes present in isolates that contained at least one ESBL gene. Non-commercial poultry isolates had the highest amount of detected *bla*_CTX-M_ genes, followed by human, commercial poultry, and water isolates. *bla*_TEM_ genes were the second-most common *bla* gene detected, with all sample types having isolates that carried these genes. Additionally, non-commercial poultry isolates were the only reservoir type that had isolates detected from each *bla* gene group analyzed (*bla*_CTX-M_, *bla*_OXA_, *bla*_SHV_, *bla*_TEM_) (Table 2).

**Table 2:** Prevalence of beta-lactamase resistance genes among sequenced third-generation cephalosporin-resistant *E. coli* (3GCR-Ec) isolates (*n* = 40), stratified by sample type and market location*.

	CTX-M^a^	OXA^b^	SHV^c^	TEM^d^
	*n (%)*	*n (%)*	*n (%)*	*n (%)*
Total	37	1	2	20
*Sample type*				
Non-commercial poultry (*n* = 16)	14 (37.9)	1 (100.0)	1 (50.0)	8 (40.0)
Commercial poultry (*n* = 8)	8 (21.6)	0 (0.0)	0 (0.0)	3 (15.0)
Human (*n* = 12)	11 (29.7)	0 (0.0)	1 (50.0)	7 (35.0)
Water (*n* = 4)	4 (10.8)	0 (0.0)	0 (0.0)	2 (10.0)
*Market*				
San Martín Jilotepeque (*n* = 18)	17 (46.0)	0 (0.0)	1 (50.0)	7 (35.0)
Guatemala City (*n* = 5)	5 (13.5)	0 (0.0)	0 (0.0)	3 (15.0)
Santa Catarina Pinula (*n* = 17)	15 (40.5)	1 (100.0)	1 (50.0)	10 (50.0)

*Many isolates had more than one ARG found.

^a^CTX-M (cefotaximase-Munich) genes.

^b^OXA (oxacillinase) genes.

^c^SHV (sulfhydryl variable) genes.

^d^TEM (Temoniera) genes.

**Figure 1:**
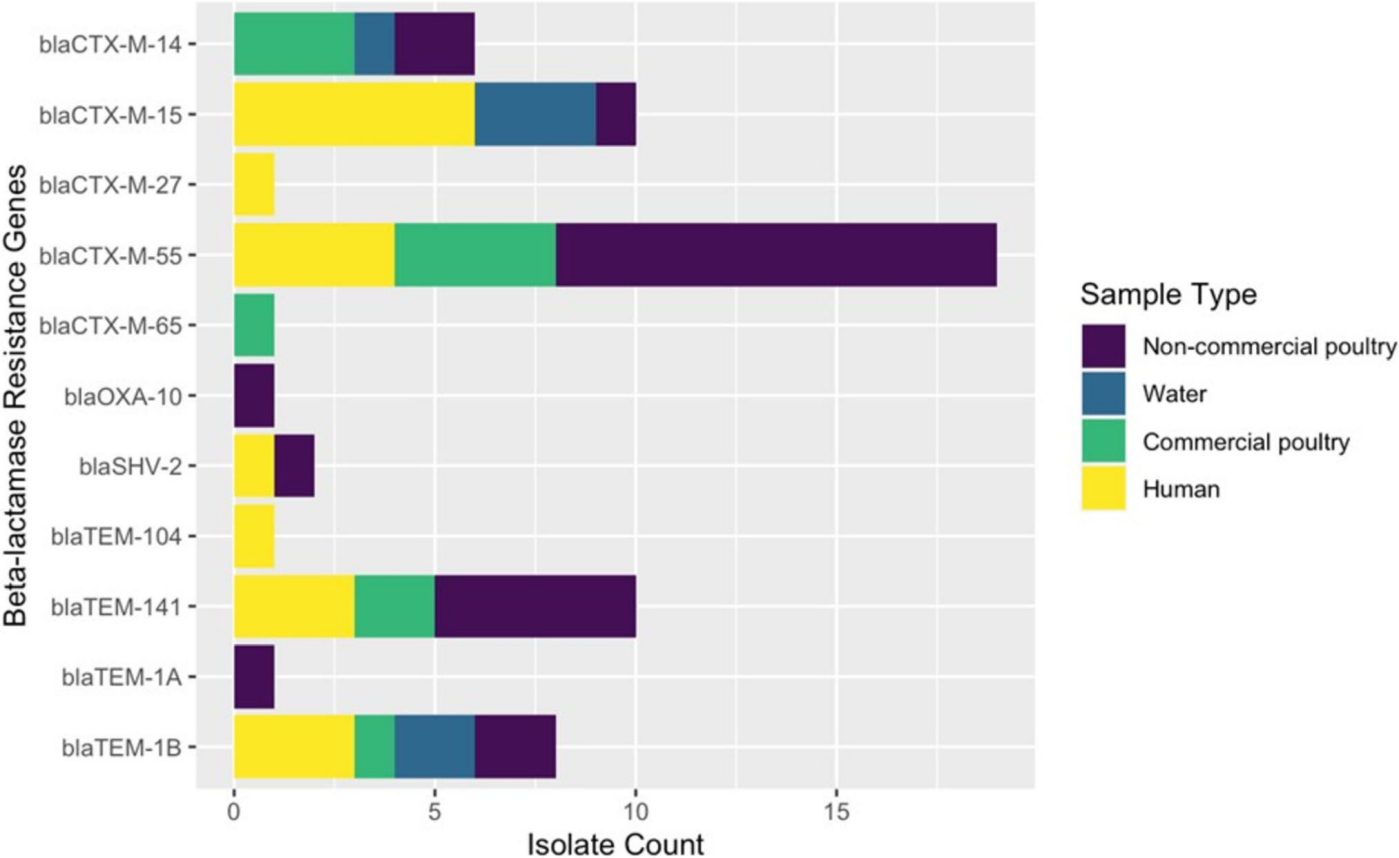
Frequency of allelic variants of ESBL genes identified in third-generation cephalosporin-resistant *Escherichia coli* (3GCR-Ec) isolates from different sample types.

*bla*_CTX-M-55_ was the most frequently detected allele among the isolates, found in the highest number of isolates and from isolates selected from non-commercial poultry, human feces, and commercial poultry (Fig. 1). Certain genes were only found in certain reservoir types. For example, *bla*_TEM-1A_, *bla*_OXA-10_, and *bla*_SHV-2_ were alleles only detected in non-commercial poultry, and *bla*_TEM-1B_, *bla*_SHV-2_, *bla*_TEM-104_, and *bla*_CTX-M-27_ were only carried by human isolates (Fig. 1).

**Figure 2:**
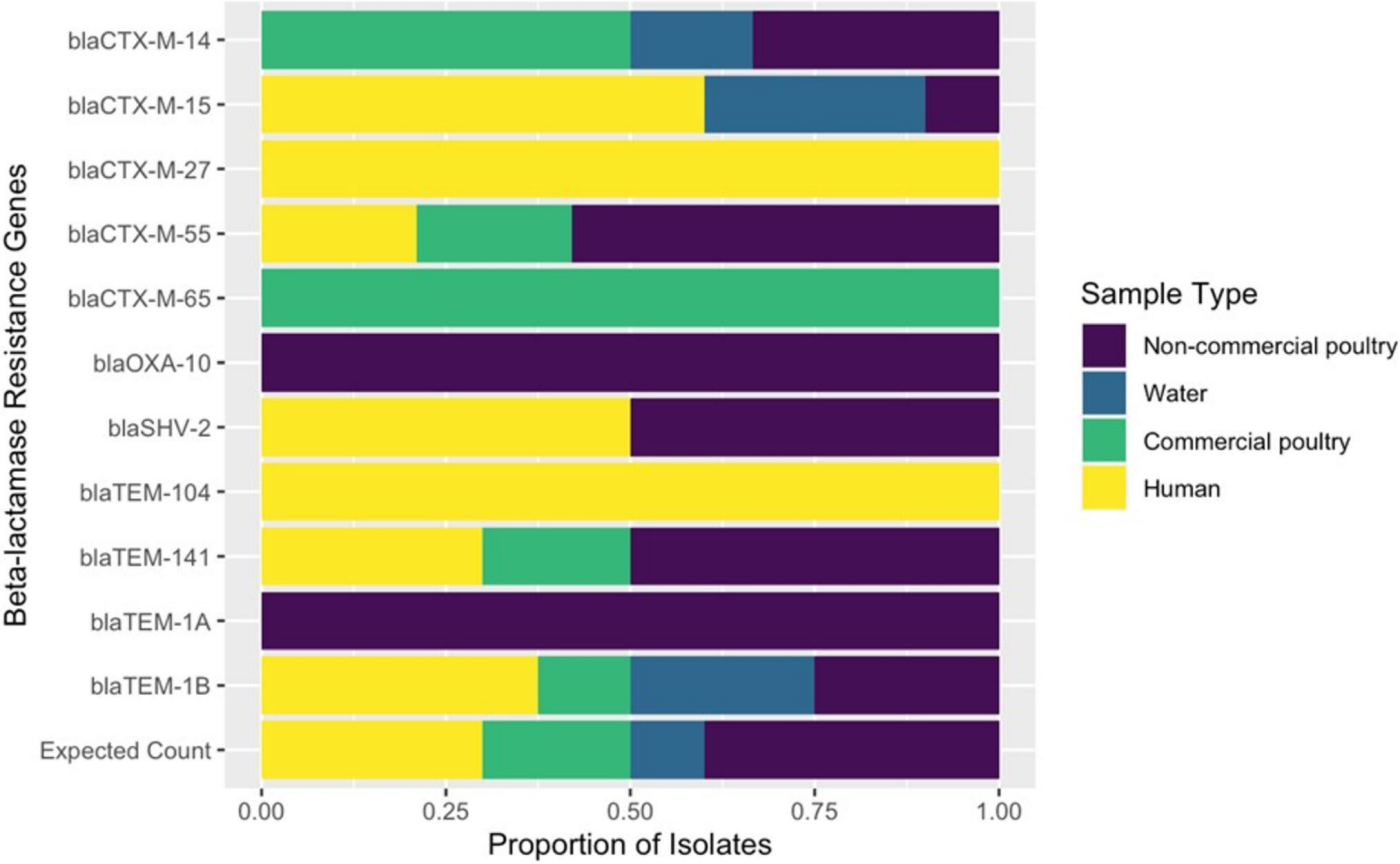
Proportion of allelic variants of ESBL genes in third-generation cephalosporin-resistant *Escherichia coli* (3GCR-Ec) isolates from sampling sites. The expected count based on the sample size of each sample type is in the bottom row.

A reference distribution was created to demonstrate how the number of isolates from each sampling type would be represented if the number of isolates per sample type was proportional to the amount found in each *bla* gene (Fig. 2). We found that the number of observed isolates was not proportional to the sample size within each sampling type. For example, only 30% of all isolates were from humans, but these represented 100% of the *bla*_TEM-104_ gene, and no human isolates carried the *bla*_OXA-10_ gene.

The only gene shared among all four sources was *bla*_TEM-1B_ (Fig. 1). However, certain *bla* genes were detected in three of the four sample types. *bla*_TEM-141_ was found in non-commercial poultry, commercial poultry, and human fecal samples. *bla*_CTX-M-15_ was found in non-commercial poultry, water, and human fecal isolates. *bla*_CTX-M-14_ was found in non-commercial poultry, commercial poultry, and water samples (Fig. 1).

### Detection of Clinically Important Sequence Types

**Figure 3:**
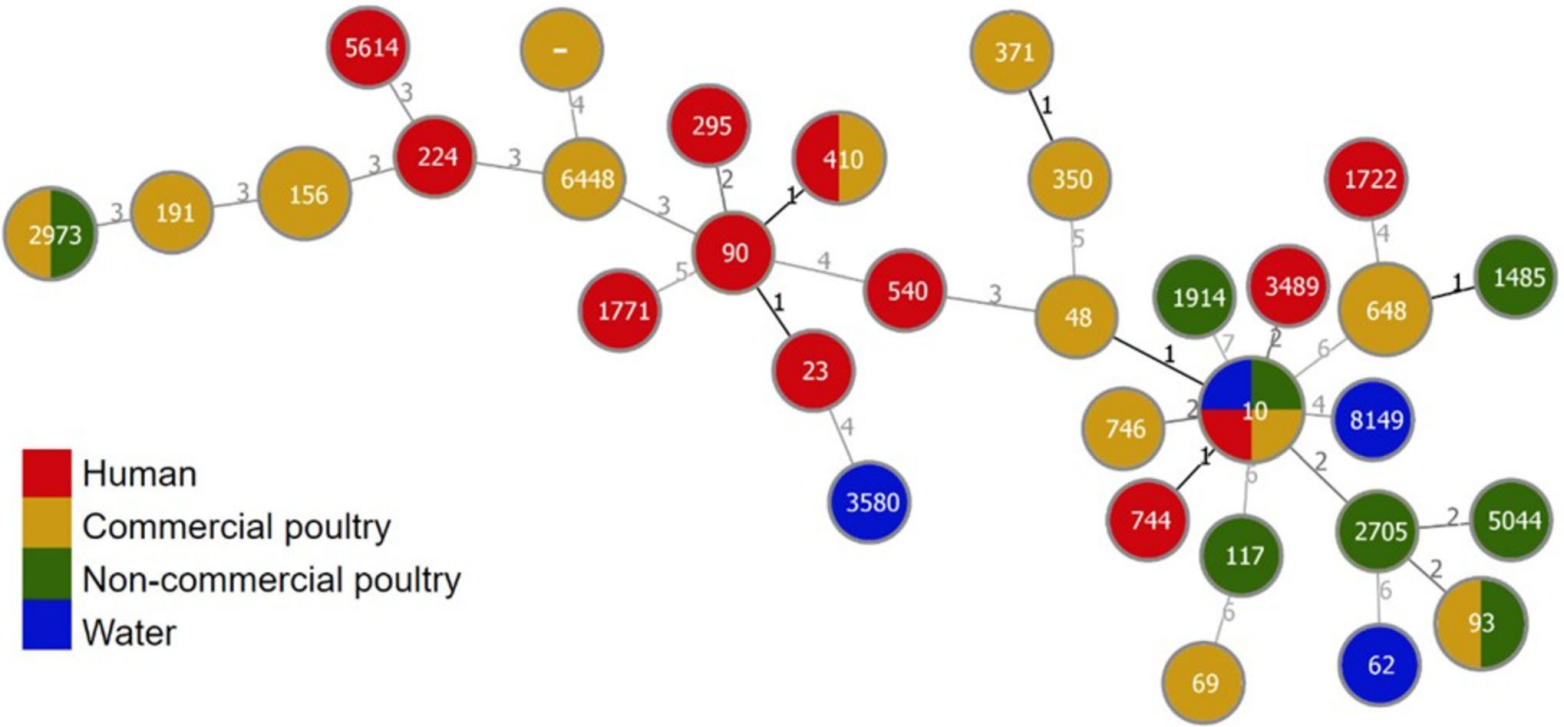
Minimum spanning tree analysis of 40 third-generation cephalosporin-resistant *Escherichia coli* (3GCR-Ec) isolates based on Multi-Locus Sequence Type (MLST) profile (number inside each circle) and according to source (color of the circles, represented by pie chart when multiple sources were present). The size of the circle indicates the relative prevalence of the isolate. Line weight (numbered) indicates phylogenetic distances. A hyphen (-) indicates an undefined sequence type.

Among the 40 isolates that contained an ESBL gene, there were 31 known *E. coli* sequence types and one undefined *E. coli* sequence type. The most common sequence type was ST10, appearing in human, commercial poultry, non-commercial poultry, and water isolates. There were only 3 other sequence types comprised of isolates from multiple sources: ST2973, ST93, and ST410. ST2973 was shared between non-commercial poultry and commercial poultry isolates. ST93 was detected only in non-commercial poultry and commercial poultry isolates as well. ST410 was found among commercial poultry and human isolates (Fig. 3).

The diversity of sequence types varied between sources. Commercial poultry isolates had the highest number of distinct STs (*n* = 14), followed by human isolates (*n* = 12 STs), non-commercial poultry isolates (*n* = 7 STs), and water isolates (*n* = 4 STs). The human and water samples had different sequence types between each of the isolates, and no sequence types were shared between isolates.

The identified sequence types known to be pandemic lineages (i.e., sequence types frequently associated with infections globally) included ST10 (*n* = 4), ST648 (*n* = 2), ST410 (*n* = 2), ST69 (*n* = 1), ST23 (*n* = 1), ST117 (*n* = 1), ST90 (*n* = 1), ST224 (*n* = 1) (see Supplemental Table S2).

## Discussion

Our study showed that one-third of the human samples contained *E. coli* resistant to third-generation cephalosporins. The study findings indicated that non-human sources of 3GCR-EC may play an important role in the dissemination of drug resistance in surveyed markets. The high detection rate of 3GCR-EC identified in non-commercial poultry may indicate that these products have an elevated chance of containing antibiotic-resistant *E. coli,* and this presents a potential target for further investigation. The high prevalence of 3GCR-EC among non-commercial poultry is also concerning, given recent studies have shown that *E. coli* from poultry is associated with the development of UTIs in humans (Aziz et al., 2024).

This study had various limitations. Our study was comprised of three markets with a limited geographic range that may not be representative of all Guatemalan markets. Additionally, our identification of non-large-scale industry chickens was dependent on evident branding and asking market vendors about the origin of the product. Due to the subjective nature of this categorization, there is a possibility that there may have been some misclassification for non-large-scale chickens. We were also unable to complete susceptibility testing on the isolates and were limited to using phenotypic testing with a third-generation cephalosporin to determine the presence of 3GCR-EC. Additionally, the presence of the beta-lactamase genes does not necessarily mean that the genes are being expressed. Though there are other pathogens associated with antibiotic resistance, the scope of this study was only focused on *E. coli*. Finally, our study had a small sample size of isolates from different sources. These limitations should be taken into account when interpreting the results of the study.

We showed that there is an overlap in the resistance genes identified across different sample types. Eleven unique ESBL genes were identified among the reservoir types and humans. Differences were displayed in the genes present between environmental sources and humans, with certain genes only detected in one reservoir type or humans. The most prevalent antibiotic resistance gene among isolates was *bla*_CTX-M-55_. There has been a documented spread of *bla*_CTX-M-55_-positive *E. coli* around the world, with China having a particularly high prevalence, where clonal transmission was frequently found between humans, animals, and the environment (Yang et al., 2023). The endemic nature of *bla*_CTX-M-55_ and its propensity to be transmitted to humans make it a resistance gene of particular concern in terms of surveillance of antibiotic resistance. The second-most prevalent gene and the gene most frequently detected among human fecal samples was *bla*_CTX-M-15_. *bla*_CTX-M-15_ has been found worldwide and is one of the most common genotypes among CTX-M beta-lactamase genes associated with drug-resistant UTIs (Pitout & Laupland, 2008).

This study illuminated potential transmission pathways of antibiotic resistance genes by identifying shared genes between reservoir types and humans. The genes carried by multiple reservoir types included *bla*_TEM-1B_, *bla*_TEM-141_, *bla*_CTX-M-14_, *bla*_CTX-M-15_, and *bla*_CTX-M-55_. These ESBL genes were most frequently found among human samples and were also detected in water and non-commercial poultry. This indicates that water and non-commercial poultry may potentially be important sources of exposure to 3GCR-EC in humans within the surveyed markets.

This study confirmed the presence of antibiotic resistance across a diverse set of reservoirs. These findings are in alignment with similar studies in low- and middle-income countries. For example, in Nairobi, Kenya, a large amount of ABR carriage in humans and livestock was shown, with 47.6% of study isolates displaying resistance to three or more antibiotic classes (Muloi et al., 2019). A study based in Antananarivo, Madagascar, detected a prevalence ranging from 17 to 42% for ESBL-EC in water, human, and animal sources (Gay et al., 2023). *bla*_CTX-M_ carrying E. *coli* were present among livestock and humans in Hanoi, Vietnam (Nguyen et al., 2021).

There are no studies to our knowledge that assess the prevalence of antibiotic resistance in multiple reservoirs in Guatemala. There has been detection of community-acquired antimicrobial-resistant Enterobacteriaceae (CA-ARE) studied separately in humans, animals, and the environment in Central America (O’Neal et al., 2020). It has also been shown that in western Guatemala, the prevalence of colonization with extended-spectrum cephalosporin-resistant Enterobacterales was 46% among individuals in the community and 67% among hospitalized patients (Ramay et al., 2023).

## Conclusion

Here, we provided evidence of antibiotic resistance across diverse sample types in community markets, with non-commercial poultry being of particular concern. We also found that many ESBL genes are shared across different sequence types and reservoir types, indicating that horizontal gene transfer could be taking place between bacterial populations from the environment, livestock, and humans. Given the exploratory data found in this study, there is a demonstrated need to further characterize transmission of antibiotic resistance through future studies with a “One Health” scope in peri-urban areas of Guatemala.

## Supplementary Information

Below is the link to the electronic supplementary material.

Supplementary file1 (DOCX 17 kb)
