# Supplementary material for: Diverse Reservoirs of Third-Generation Cephalosporin-Resistant Escherichia coli in Guatemala
Source: Ecohealth. 2026 Jan 23;23(2):223–32. doi: 10.1007/s10393-026-01775-9 (PMC13287249; doi:10.1007/s10393-026-01775-9)
Supplement: Supplementary file 1 — Supplementary file1 (DOCX 17 kb) [file 10393_2026_1775_MOESM1_ESM.docx]

Supplemental Materials

**Diverse reservoirs of third-generation cephalosporin-resistant *Escherichia coli***

**in Guatemala**

**Supplemental Table 1.** Distribution of isolate characteristics stratified by sample type and market, among environmental samples from peri-urban and urban markets in Guatemala City, Guatemala.

|  | **No. Isolates** | **(%)** |
| --- | --- | --- |
| *Total* | 40 |  |
| *Sample Type* |  |  |
| Non-commercial poultry | 16 | (40.0) |
| Commercial poultry | 8 | (20.0) |
| Produce | 0 | (0.0) |
| Human | 12 | (30.0) |
| Water | 4 | (7.8) |
| *Market* |  |  |
| San Martín Jilotepeque | 18 | (49.0) |
| Guatemala City | 5 | (9.8) |
| Santa Catarina Pinula | 17 | (41.2) |

**Supplemental Table 2.** Prevalence of clinically important sequence types (ST) among sequenced 3GCR-EC isolates (n=40) from environmental samples *(Manges et al., 2019).*

| **ST No. Isolates (%)** |
| --- |
| 10 4 (10.0)  648 2 (5.0)  410 2 (5.0)  69 1 (2.5)  23 1 (2.5)  117 1 (2.5)  90 1 (2.5)  224 1 (2.5) |

3GCR-EC: third-generation cephalosporin-resistant *E. coli.*
